# Optimization of Production Parameters for Andrographolide-Loaded Nanoemulsion Preparation by Microfluidization and Evaluations of Its Bioactivities in Skin Cancer Cells and UVB Radiation-Exposed Skin

**DOI:** 10.3390/pharmaceutics13081290

**Published:** 2021-08-18

**Authors:** Rathapon Asasutjarit, Nawarat Sooksai, Adryan Fristiohady, Kriyapa Lairungruang, Shiow-Fern Ng, Asira Fuongfuchat

**Affiliations:** 1 Thammasat University Research Unit in Drug, Health Product Development and Application (DHP-DA), Department of Pharmaceutical Sciences, Faculty of Pharmacy, Thammasat University, Pathum Thani 12120, Thailand; nawarat.sook@dome.tu.ac.th (N.S.); adryanfristiohady@uho.ac.id (A.F.); 2 Department of Pharmacology and Clinical Pharmacy, Faculty of Pharmacy, Universitas Halu Oleo, Kendari 93132, Indonesia; 3 Department of Applied Thai Traditional Medicine, Praboromarajchanok Institute, Abhaibhubejhr College of Thai Traditional Medicine Prachinburi, Prachinburi 25230, Thailand; kriyapa@acttm.ac.th; 4 Centre for Drug Delivery Technology, Faculty of Pharmacy, University Kebangsaan Malaysia, Jalan Raja Muda Abdul Aziz, Kuala Lumpur 50300, Malaysia; nsfern@ukm.edu.my; 5 National Metal and Materials Technology Center, National Science and Technology Development Agency (NSTDA), Thailand Science Park, Pathum Thani 12120, Thailand; asiraf@mtec.or.th

**Keywords:** andrographolide, nanoemulsion, microfluidization, design of experiment, central composite design, optimization, transdermal drug delivery, skin cancer, tyrosinase inhibitor, UVB irradiation

## Abstract

Andrographolide (AG) is an active compound isolated from *Andrographis paniculata* (Family Acanthaceae). Although it possesses beneficial bioactivities to the skin, there is insufficient information of its applications for treatment of skin disorders due to low water solubility leading to complications in product development. To overcome the problem, an AG-loaded nanoemulsion (AG-NE) was formulated and prepared using a microfluidization technique. This study aimed to investigate the effect of pressure and the number of homogenization cycles (factors) on droplet size, polydispersity index and zeta potential of AG-NE (responses) and to determine the effect of AG-NE on skin cancer cells and UVB irradiation-induced skin disorders in rats. Relationships between factors versus responses obtained from the face-centered central composite design were described by quadratic models. The optimum value of parameters for the production of optimized AG-NE (Op-AG-NE) were 20,000 psi of pressure and 5 homogenization cycles. Op-AG-NE showed promising cytotoxicity effects on the human malignant melanoma- (A375 cells) and non-melanoma cells (A-431 cells) via apoptosis induction with a high selectivity index and also inhibited intracellular tyrosinase activity in the A375 cells. Op-AG-NE could reduce melanin index and healed UVB irradiation exposed skin. Op-AG-NE thus had potential for treatment of skin cancers and skin disorders from exposure to UVB radiation.

## 1. Introduction

Andrographolide (AG) is one of diterpenoids isolated from stems and leaves of *Andrographis paniculata* (Family Acanthaceae), a medicinal plant used as an ingredient in remedies for fever, cold, sore throat and diarrhea in China, Japan, India, Southeast Asia and Europe [1,2]. To date, the pharmacological activities of AG have been extensively investigated. This terpene-based compound exhibited strong bioactivities for treatment of various diseases, for example, diseases related to inflammation [2], viral and bacterial infection [1], central nervous system disorders [3], including cancers, e.g., leukemia, breast cancer and non-melanoma [1,4,5]. Recently, it has been found to be beneficial to the skin. The previous studies reported that the methanolic extract of *Andrographis paniculata*, containing AG as a major bioactive compound could protect keratinocytes from oxidative stress [5]. Its water-soluble derivative, AG sodium bisulfite, could prevent skin photoaging in mice [6]. Moreover, it had promising activities for suppressing melanin synthesis in mice exposed to ultraviolet (UV) radiation [7]. AG has thus attracted attention for skin disorder management, particularly skin pigmentation, skin cancer and skin damage from UV radiation. 

Skin pigmentation is generally determined by extent and type of epidermal melanin, i.e., pheomelanin and eumelanin, which provide red/yellow and brown/black skin color, respectively [8]. Melanocytes are the cells responsible for melanogenesis. Melanogenesis is started in melanosomes within melanocytes via modification of tyrosine into dihydroxyphenylalanine (DOPA) and dopaquinone, with tyrosinase as an oxidative enzyme [8,9]. Skin pigmentation directly depends on the number, cellular distribution and type of melanosomes in the epidermis, which are influenced by intrinsic factors, i.e., genetics and skin types [10]. The extrinsic factors, for example, UV radiation, environmental pollutions, and degree of sunlight exposure, also affect the skin pigmentation [9]. These latter factors are considered as crucial correlating factors of skin damage and susceptibility to skin cancers [6,7,11]. In particular, in intermittent and long-term exposure to UV radiation or sunlight, they not only darken the skin by increasing melanogenesis, but also cause melanoma and non-melanoma skin cancer. They can induce skin cancers via reduction of cell-mediated immune responses, production of reactive oxygen species (ROS) and alteration of DNA [11]. 

Nowadays, some natural compounds possessing skin-protective effects as well as melanogenesis suppressive activity have been added into skin-care formulations and already commercialized [7,9]. However, there is insufficient information of application of AG for treatment of skin disorders such as skin cancers, UVB-induced skin pigmentation and skin damage. This might be due to low water solubility of AG (around 0.10 mg/mL) [12], leading to poor bioavailability, low efficacy [6,12] and complications in product development. It thus requires suitable solubilizers and carriers for transdermal delivery. 

Nanoemulsions (NEs) are a type of emulsion containing submicron droplets of an internal phase (around 50–500 nm in diameter) dispersed in an external phase [13]. NEs have advantages over conventional emulsions due to their small droplets leading to efficient cellular uptake by endocytosis, and henceforth increase drug absorption. The submicron size of the droplets also decreases the possibility of creaming and sedimentation induced by gravity and thus improves product stability. Importantly, oil in water (*o*/*w*) NEs could improve the solubility of lipophilic drugs by the facilitation of emulsifiers and oil droplets dispersed in the aqueous phase [14,15]. Since NEs are thermodynamically unstable, they need energy for droplet formation [13]. In the present study, the microfluidization technique, a high-energy emulsification technique, was used for the preparation of a nanoemulsion (NE) containing AG (AG-NE). This technique generates efficient energy for the creation of submicron droplets through the collision of two flowing streams of a mixture of oil- and water phase in the interaction chamber under a high pressure of a microfluidizer. It can be applied for the production of NEs on both the laboratory-scale and industrial scale [16]. More importantly, microfluidization technique is convenient to scale up, i.e., the same production conditions, such as pressure and number of homogenization cycles can be applied [17]. Production of NEs by a microfluidizer requires efficient energy to generate small and homogeneous droplets. However, coalescence of newly formed droplets is always found when the supplied energy is in excess. Consequently, determination of optimum values of pressure and the number of homogenization cycles of the microfluidization is necessary to obtain the robust production process of NEs. Due to fewer data on the production of AG-NEs, especially for transdermal applications of AG, this study is necessary. 

The objectives of this study were (1) to investigate the effect of pressure and number of homogenization cycles on droplet size, PDI and zeta potential of AG-NEs prepared by microfluidization technique; (2) to determine cytotoxicity of AG-NEs to the normal skin cells and skin cancer cells; and (3) to determine the effect of AG-NE on UVB irradiation-induced skin pigmentation and skin damage.

## 2. Materials and Methods

### 2.1. Materials

Andrographolide (AG) with a purity of more than 98% was supplied by TCI (Tokyo, Japan). Fetal bovine serum (FBS), phosphate-buffered saline (PBS) (pH 7.4 and 7.0), and cell culture media were obtained from Gibco (Amarillo, TA, USA). Dimethyl sulfoxide, 3-(4,5-dimethy-2-thiazolyl)-2,5-diphenyl-2*H*-tetrazolium bromide (MTT), propylene glycol, polysorbate 80, soybean lecithin (called briefly as lecithin), a cellulose dialysis tube were contributed by Merck (Darmstadt, Germany). AnnexinV-FITC, binding buffer and propidium iodide were supplied by BD Biosciences (Franklin Lakes, NJ, USA). Coconut oil, jojoba oil, and sesame oil were obtained from Hong Huat (Bangkok, Thailand). All other chemicals and solvents were analytical grade and were used as received. 

### 2.2. Preparation of Andrographolide-Loaded NEs (AG-NEs) and Blank NE

The formulation of AG-NEs used in this study was based on the formulation of AG-NEs, which was previously reported with some modifications [4]. All AG-NEs contained the same compositions and were prepared by microfluidization technique. However, their preparation process was varied in pressure and number of homogenization cycles. Briefly, AG (0.1 g) was dispersed in a mixture of the oils that are beneficial to the skin: coconut oil, sesame oil, and jojoba oil at a weight ratio of 2:1:1 (20 g), which provided the highest solubility of AG when compared to other ratios of these oils. Thereafter, Tween 80 and lecithin at a weight ratio of 4:1 (10 g), ethanol and propylene glycol at a weight ratio of 1:1 (5 g) and paraben concentrate (0.1 g) were added and mixed thoroughly until a clear solution was obtained. Subsequently, purified water was added up to 100 g. The obtained mixture was mixed by a high-speed homogenizer (Ultraturrax T8, IKA-Werke GmbH & Co., Staufen, Germany), at 1000 rpm for 10 min to make a pre-emulsion. Finally, the pre-emulsion was homogenized via a microfluidizer (LM20, Microfluidics, Westwood, MA, USA) at various levels of pressure and number of homogenization cycles according to the face-centered central composite design (FCCD) shown in Table 1 [18]. A NE base containing the same compositions as all AG-NEs without addition of AG was also prepared using the same pressure and number of homogenization cycles for the production of an optimized AG-NE that possessed an optimum value of droplet size, PDI and zeta potential. 

The NE base and optimized AG-NE will be presented as Blank NE and Op-AG-NE, respectively, throughout this article.

### 2.3. Determination of Effects of Pressure and Number of Homogenization Cycles on Droplet Size, PDI and Zeta Potential of AG-NEs 

To elucidate the effect of microfluidization condition on droplet size, PDI and zeta potential of AG-NEs, the FCCD was chosen for planning the experiment. The reasons for choosing the FCCD were that this design needs fewer experimental runs than those of the full factorial design. It adds center points and axial points to the initial factorial design, leading to the experimental data fitting a quadratic model properly [18,19]. Once the model is obtained, it can be used for the prediction of certain responses at known values of factors. From the preliminary experiments, two production parameters, i.e., pressure and number of homogenization cycles, were critical factors for droplet size, PDI and zeta potential of AG-NEs. These two parameters were thus used as factors for the FCCD. 

Twelve experiments containing 4 factorial points, 4 axial points and a center point with 4 replications for the estimation of an error were generated and analyzed by the statistical software (Design-Expert V. 13, StatEase Inc., Minneapolis, MN, USA). AG-NE numbers representing the experimental runs, actual values and coded values of factors at various levels are presented in Table 1. For the regression coefficients, a test of significance was conducted to obtain the regression equations including only the terms with statistical significance. A statistically significant *F*-ratio (*p*-value < 0.05) and adjusted determination coefficients (Adj-R^2^) between 0.8–1.0, which related to non-statistically significant lack of fit (*p*-value > 0.05), were the criteria for model selection [18,19]. The effect of the factors on the responses were depicted as response surface plots.

### 2.4. Determination of Droplet Size, PDI, Zeta Potential and pH

Droplet size and PDI of AG-NEs and Blank NE were measured by dynamic light scattering technique via Zetasizer (Malvern Instrument Nano-ZS, Malvern, UK). Their zeta potential was determined by an electrophoretic light scattering technique by the same instrument. The pH values of AG-NEs and Blank NE were measured by a pH meter (Mettler-Toledo, Greifensee, Switzerland). 

### 2.5. Entrapment Efficiency and Drug Loading Capacity Measurement of AG-NEs

Entrapment efficiency (EE) and loading capacity (LC) of a representative AG-NE were determined after sample preparation by a stirred ultrafiltration cell (10 kDa MWCO) (Merck, Germany). The filtrate was analyzed for free AG content by high performance liquid chromatography (HPLC) technique. EE and LC of AG-LNE were calculated according to Equations (1) and (2), respectively.

(1) $$\mathrm{EE}\;(\%)=\frac{\mathrm{total}\;\mathrm{amount}\;\mathrm{of}\;\mathrm{loaded}\;\mathrm{AG}-\mathrm{amount}\;\mathrm{of}\;\mathrm{free}\;\mathrm{AG}}{\mathrm{total}\;\mathrm{amount}\;\mathrm{of}\;\mathrm{loaded}\;\mathrm{AG}}\times \;100\;$$

(2) $$\mathrm{LC}\;(\%)=\frac{\mathrm{total}\;\mathrm{amount}\;\mathrm{of}\;\mathrm{loaded}\;\mathrm{AG}-\mathrm{amount}\;\mathrm{of}\;\mathrm{free}\;\mathrm{AG}}{\mathrm{total}\;\mathrm{amount}\;\mathrm{of}\;\mathrm{oils}\;\mathrm{and}\;\mathrm{emulsifiers}}\times \;100\;$$

### 2.6. Determination of AG Content in AG-NEs

A representative AG-NE (0.05 g) was dissolved in the mobile phase (5 mL) and then vortexed for 5 min prior to being filtered through a 0.45-μm membrane. AG content in AG-NE was analyzed by HPLC technique according to the analysis protocol described in the following section.

### 2.7. Analysis of AG Content by HPLC Technique

The content of AG in a representative AG-NE and all samples were quantified by HPLC technique. The analysis was performed by an HPLC instrument (Shimadzu-SPD-20A, Kyoto, Japan) via a C18 column (15 cm × 4.6 mm) with particle size of 5 μm (Zorbax eclipse, Agilent, Santa Clara, CA, USA). Twenty microliters of the samples were injected into the HPLC system. They were eluted by the mobile phase, which consisted of methanol, acetonitrile and water (65:20:15) at a flow rate of 1.5 mL/min. A uv-vis detector was set at 223 nm. 

### 2.8. Morphology Observation of AG-NEs and Blank NE

The morphology of a representative AG-NE and Blank NE were observed by using a transmission electron microscopy (TEM) technique. The samples were diluted and dropped on a copper grid coated with carbon film. Thereafter, they were stained with 1% *w*/*v* uranyl acetate solution and dried in an ambient condition. Micrographs of the samples were taken by using a JEM-2100 transmission electron microscope (JEOL Ltd., Tokyo, Japan) at 100 kV. 

### 2.9. FT-IR Spectroscopy Analysis 

To determine interactions between AG and NE base, Fourier transform infrared (FT-IR) spectra of AG, Blank NE and a representative AG-NE were determined by using an FT-IR spectrometer (PerkinElmer Spectrum One, Waltham, MA, USA). Prior to the analysis, Blank NE and a representative AG-NE were dried using the freeze-drying technique via an Eyela FD-1 freeze dryer (Eyela, Kyoto, Japan) for 24 h with no additives. Each sample was ground and mixed together with KBr powder at a ratio of 1:100. Then, it was pressed into pellets. The signal averages were obtained for 32 scans with a four cm^−1^ resolution from 4000 to 600 cm^−1^.

### 2.10. In Vitro Drug Release and Skin Permeation Study of AG-NEs

The release kinetic of AG from a representative AG-NE was investigated by using modified Franz diffusion cells. AG-NE (0.4 g) was applied on cellulose acetate membrane (12 kDa MWCO), which was placed between the donor and receptor compartment. The receptor compartments were filled with PBS (pH 7.4) containing 10% *v*/*v* ethanol as a receiving medium and was maintained at 37 ± 1 °C. A twelve-milliliter sample of the receiving medium was withdrawn at 5, 10, 15, 30, 60 min, then again at every hour up to 10 h. It was replaced with an equal volume of fresh medium. The withdrawn samples were analyzed for the content of AG by an HPLC technique in triplicate.

A skin permeation study of a representative AG-NE was performed by using modified Franz diffusion cells. The skin used in this study was obtained from newborn pigs which had naturally died after birth and were supplied by a local pig farm in Pathum Thani Province, Thailand. Subcutaneous fat was removed and cut into a size of 2 × 2 cm, which could cover the diffusion area (1.767 cm^2^). An hour prior to the study, the pig skin was equilibrated in PBS (pH 7.4) at 37 °C. AG-NE was applied on the pig skin that was placed on the receptor unit containing ethanol (10% *v*/*v*) in PBS (pH 7.4) as a receiving solution. The receiving solution was stirred by a magnetic stirrer with temperature control at 37 ± 1 °C. It was withdrawn at 1, 2, 3, 4, 6, 8, 10, 12, 14, 16, 18, 20, and 24 h and replaced with the fresh solution to keep the constant volume of the receiving solution. The AG content in the receiving solution was determined by the HPLC technique. A flux of AG through the pig skin was calculated based on the Fick’s first law of diffusion. The study was performed in 3 replicates.

### 2.11. Cytotoxicity Test of AG Solution and AG-NEs 

The cytotoxicity tests of AG solution, Blank NE and a representative AG-NE were performed in the human skin fibroblasts (HFF-1 cells) (SCRC-1041; ATCC, Manassas, VA, USA), the human malignant melanoma cells (A375 cells) (CRL-1375; ATCC, USA) and the epidermoid carcinoma cells (A-431 cells) (CRL-1555; ATCC, USA). They were cultured in complete Dulbecco’s Modified Eagle Medium (DMEM), at 37 °C under 5% CO_2_ atmosphere and their cell viability (CV) was determined after exposure to AG and AG-NE by MTT assay. The cells were seeded in 96-well plates with a density of 5 × 104 cells/well/100 µL. After confluence, they were incubated with the test samples dissolved in the complete medium containing 0.5% *v*/*v* DMSO (100 μL) at various concentrations based on AG concentration as listed: 1, 10, 30, 50, and 100 μg/mL. The untreated cells were used as a negative control. After a 24-h incubation period, the test samples were removed from the wells and replaced with fresh complete medium (100 μL) and MTT solution in PBS pH 7.4 (5 μg/mL) (10 μL) was added into each well and incubated with the cells for 4 h. Formazan crystal in the cells was then dissolved with DMSO (10 μL) after MTT solution was removed. An optical density (OD) of each well was measured by a microplate reader (Spectrostar Omega, BMG Labtech, Ortenberg, Germany) at a wavelength of 570 nm. CV values of these cells were calculated using Equation (3).

(3) $$\mathrm{CV}\;(\%)=\frac{{\mathrm{OD}}_{\mathrm{sample}}}{{\mathrm{OD}}_{\mathrm{control}}}\times \;100\;$$

where OD_sample_ and OD_control_ were an OD of the wells containing the cells incubated with the samples and MTT solution, and an OD of the wells containing the cells incubated with MTT solution without addition of the samples, respectively.

To investigate the cytotoxic selectivity, a selectivity index (SI) of the test samples was determined by the following Equation (4):

(4) $$\mathrm{SI}=\frac{\mathrm{IC}50\;\mathrm{of}\;\mathrm{normal}\;\mathrm{cells}}{\mathrm{IC}50\;\mathrm{of}\;\mathrm{cancer}\;\mathrm{cells}}\times \;100\;$$

where IC50 was a half-maximal inhibitory concentration of the test samples that induce fifty percentage of CV of the tested cells. 

### 2.12. Analysis of Apoptosis of the Skin Cancer Cells Induced by AG Solution and AG-NEs 

Since exposure to UV irradiation and sunlight causes not only skin pigmentation, but also skin cancers, i.e., melanoma- and non-melanoma skin cancer, this study was conducted to determine the cytotoxicity of AG solution, Blank NE and a representative AG-NE against the A375 cells and A-471 cells via apoptosis induction by a flow cytometry technique. Both cells were seeded in 12-well plates at a density of 2 × 10^5^ cells/well/1000 μL and incubated at 37 °C, under 5% CO_2_ atmosphere until confluence. They were incubated with the test samples for 24 h. After incubation, the cells were suspended in 1X binding buffer at a concentration of 105 cells/100 µL. Annexin V-FITC (4 µL) and propidium iodide (PI) (4 µL) (BD PharmingenTM, BD Biosciences, San Diego, CA, USA) were added to the cells and incubated for 15 min. Then, the binding buffer (400 µL) was added to each tube containing the cells prior to analysis by a flow cytometry instrument (BD FACSVerse™, BD Biosciences, USA) at an excitation/emission wavelength of 490/520 and 535/617 nm for FITC and PI, respectively. The fluorescent-activated cell sorting (FACS) were plotted and analyzed by BD FACSVerse™ software V. 1.0 (BD Biosciences, USA). 

### 2.13. Determination of Intracellular Tryrosinase Inhibitory Activity of AG Solution, AG-NEs, and Blank NE 

The activity of intracellular tyrosinase in the A375 cells was determined after the cells were incubated with the medium containing DMSO and the test samples as listed: AG solution, a representative AG-NE, Blank NE, or kojic acid (a positive control). The cells in 6-well plates (1 × 10^6^ cells) were exposed to these test samples at an appropriate concentration that was not toxic to the cells for 72 h. Thereafter, the cells were collected and kept in microfuge tubes. They were washed twice with PBS, then, 1% *v*/*v* Triton-X in phosphate buffer (pH 6.8) (200 μL) was added into the microfuge tubes with vigorous mixing. The cell extracts were separated by centrifugation at 12,000 rpm, 4 °C for 10 min. The supernatant was subjected to the test for measurement of intracellular tyrosinase activity of the A375 cells. PBS (pH 6.8) containing 0.2% *w*/*v* L-DOPA (50 μL) was put in the each well of 96-well plates containing the cell extracts (100 μL). They were mixed and incubated at 37 °C for 0.5 h. The plates were subjected to read the OD at a wavelength of 490 nm by using the microplate reader [7]. Tyrosinase inhibitory activity of the test samples was determined by the following Equation (5):

(5) $$\mathrm{Tyrosinase}\;\mathrm{inhibitory}\;\mathrm{activity}\;(\%)=\frac{{\mathrm{OD}}_{C}-{\mathrm{OD}}_{T}}{{\mathrm{OD}}_{C}}\times \;100\;$$

where OD_C_ and OD_T_ were an OD of the wells containing the extract from the cells without exposure to the test samples, and an OD of the wells containing the extract from the A375 cells incubated with the test samples, respectively.

### 2.14. Investigation of Effect of AG-NEs on UVB Irradiation-Induced Skin Pigmentation and Skin Damage in Rats

This in vivo study was performed to determine the effect of AG-NE on skin pigmentation and skin damage in rats induced by UVB irradiation. The viscosity of a representative AG-NE and Blank NE used in this study were modified by addition of methylcellulose into the formulation to improve their adhesiveness. 

A representative AG-NE and Blank NE consisting of methylcellulose (M0387, Sigma-Aldrich, St. Louis, MO, USA) (2% *w*/*w*) was prepared. The viscosity and flow behavior of AG-NE was investigated by a steady-shear sweep test at 25 ± 0.1 °C via a controlled-strain rotational rheometer (ARES G2, TA Instrument, New Castle, DE, USA) with titanium parallel plates (50 mm in diameter). The test was set at 1 s^−1^ and 300 s^−1^ for the initial- and final shear rate, respectively.

Forty male Wista rats (10–12 weeks old, 250–350 g) were obtained from the Laboratory Animal Research Unit, Faculty of Veterinary Medicine, University Kebangsaan, Malaysia and were divided equally into four groups (10 rats/group). Group I: naïve rats, rats neither exposed to UVB radiation nor received the test samples; Group II: sham rats, they were the UVB-exposed rats those did not receive any test samples; Group III: UVB-Blank NE gel rats, they were the UVB-exposed rats receiving Blank NE gel; and Group IV: UVB-Op-AG-NE gel rats, they were the UVB-exposed rats receiving Op-AG-NE gel. The protocol for use and care of animals for this study was approved by the University Kebangsaan Malaysia Animal Ethics Committee (UKMAEC No.: FF/2019/SHIOWFERN/24-APRIL/100 6-MAY-2019-FEB-2020) on 24 April 2019. The experiment was conducted at Faculty of Pharmacy, University Kebangsaan, Malaysia. It was in full compliance with local, national, ethical, and regulatory principles and local licensing regulations, per the spirit of Association for Assessment and Accreditation of Laboratory Animal Care (AAALAC) International’s expectations for animal care and use/ethics committees. 

After acclimatization for 7 days, the rats were subjected to the experiment. Naïve rats in Group I did not receive any treatments during the experiment period. Meanwhile, sham rats (Group II), UVB-Blank NE gel rats (Group III) and UVB-Op-AG-NE gel rats (Group IV) were continuously exposed to UVB radiation with an intensity of 400 mJ/cm 2 for 30 min/day, since day 1 to day 14. During this period, UVB-Blank NE gel rats and UVB-Op-AG-NE gel rats received 0.2 g of Blank NE gel and Op-AG-NE gel that contained 0.1% *w*/*w* AG, respectively, 1 h before exposure to UVB radiation. The gels were applied on the dorsal skin (4.0 cm × 5.0 cm) every day and further applied until day 21, although, UVB irradiation was stopped since day 14. On day 14 and 21, the rats in every group were measured melanin index of their skin by using a Mexameter (MX18E, Courage+Khazaka GmbH, Cologne, Germany) and visually observed macroscopic changes of the skin appearance. The rats were sacrificed on day 21 after evaluations were complete [20,21].

### 2.15. Statistical Analysis

The results were presented as a mean ± standard deviation (SD). Statistical analysis for general comparison of the treatment effects was performed by either independent t-test or one-way ANOVA with Tukey’s multiple comparisons at a significant level of 0.05. 

## 3. Results and Discussion

### 3.1. Preparation of AG-NEs 

All o/w AG-NEs prepared in this study exhibited milky characteristics. They had white color and turbidity in appearance and could flow freely when their containers were tilted. All of them were stable without phase separation after they were centrifuged at 10,000 rpm for 30 min, which was an accelerated stability test and performed immediately after they were prepared. Their droplet size, PDI, and zeta potential of AG-NEs are shown in Table 2 as observed values of the responses. They implied that all AG-NEs possessed droplet size in a nanometer range with moderately broad droplet size distribution and low negative values of zeta potential. It was also found that the values of droplet size, PDI and zeta potential of each AG-NE did not significantly change during storage at an ambient condition for 12 weeks. Therefore, all AG-NEs had good physical stabilities for at least 12 weeks. 

The results shown in Table 2 suggested that although AG-NEs contained the same ingredients, their droplet size, PDI and zeta potential were obviously different. This finding indicated the production parameters as listed: pressure and number of homogenization cycles affected droplet size, PDI and zeta potential of AG-NEs. 

### 3.2. Regression Analysis 

The results of regression analysis of the relationships between the factors (pressure and number of homogenization cycles) versus the responses (droplet size, PDI and zeta potential) showed acceptable quadratic models in Equations (6)–(8) for a description of the effects of both pressure and number of homogenization cycles on droplet size, PDI and zeta potential of AG-NEs.

Droplet size = 768.26 − 0.04(*a*) − 39.38(*b*) + 9.95 × 10^−7^ (*a*^2^) + 3.25(*b*^2^) (6)

PDI = 2.01 − (1.25 × 10^−4^)(*a*) − 0.21(*b*) + (2.86 × 10^−9^)(*a*^2^) + 0.03(*b*^2^) (7)

Zeta potential = 17.64 − (1.79 × 10^−3^)(*a*)−3.98(*b*) + (4.17 × 10^−8^)(*a*^2^) + 3.87(*b*^2^) (8)

where (*a*) and (*b*) are the pressure and number of homogenization cycles, respectively. The model *p*-value of Equations (6)–(8) after elimination of insignificant interaction term of pressure and number of homogenization cycles (*a* × *b*) (*p*-values > 0.05), were statistically significant at a *p*-value < 0.0001. They implied that there was less than a 0.01% chance that the *F*-ratios of these equations occurred from noise. The Adj-R^2^ values of Equations (6)–(8) were 0.9947, 0.9911 and 0.9676 respectively. These satisfied Adj-R^2^ values suggested that the obtained quadratic models could explain 99.47%, 99.11% and 96.76% for variability of droplet size, PDI and zeta potential, respectively, from changes of the values of pressure and number of homogenization cycles.

The *p*-values of the lack-of-fit test for Equations (6)–(8) were statistically insignificant at a *p*-value of 0.5586, 0.3706 and 0.3007, respectively, indicating that the obtained quadratic models fitted the data well. Therefore, they could describe the relationships between the factors and the responses properly, and could predict the droplet size, PDI and zeta potential of AG-NEs when the pressure and number of homogenization cycles were adjusted [18,19]. These findings were confirmed by the mean of predicted values of the responses shown in Table 2. They pointed out that all of the observed values of the responses were within 95% confidence interval of the predicted values estimated by using these quadratic models and are analyzed by the statistical software (Design-Expert V. 13). 

### 3.3. Effects of Pressure and Number of Homogenization Cycles on Droplet Size and PDI of AG-NEs

**Figure 1:**
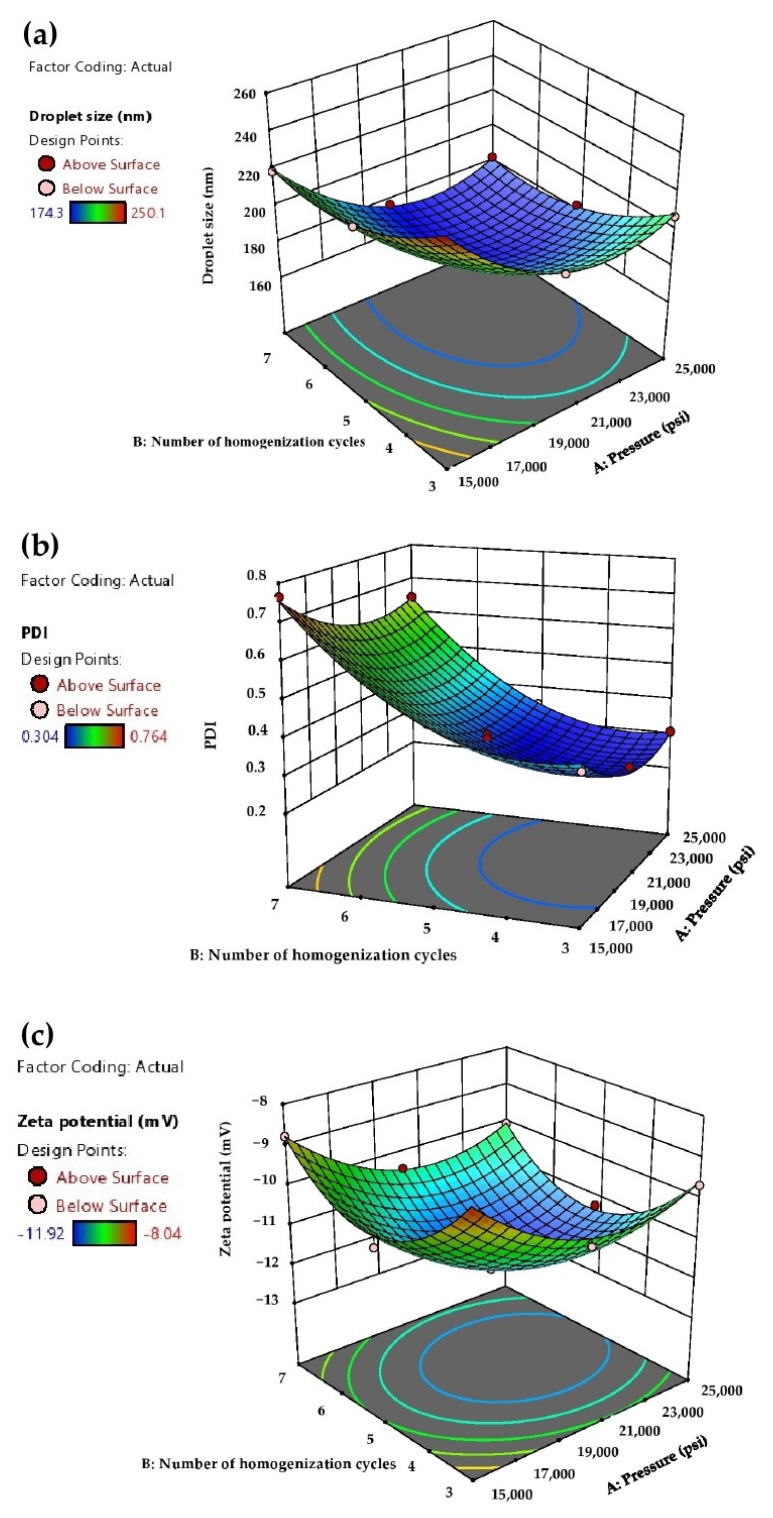
Response surface plots of the effect of pressure and number of homogenization cycles on the responses: (**a**) droplet size, (**b**) PDI and (**c**) zeta potential of AG-NEs.

The effects of pressure and number of homogenization cycles on droplet size, PDI and zeta potential of AG-NEs shown in Equations (6)–(8) were depicted as a response to the surface plots in Figure 1. Figure 1a,b show that when the pressure and number of homogenization cycles were raised from 15,000 to 20,000 psi and from 3 cycles to 5 cycles, respectively, the droplet size and PDI of AG-NEs were decreased. This finding was due to the high pressure in the microfluidizer providing a high input energy to the pre-emulsion and forced streams of the pre-emulsion to flow through a microchannel of the microfluidizer with a higher rate. A tremendous shear force was then created for the production of fine emulsion droplets [22]. In particular, when a higher number of homogenization cycles was applied, it increased the exposure time of the pre-emulsions to such high energy generated by the microfluidizer [16]. 

Unfortunately, a further increase in the pressure and number of homogenization cycles of the microfluidizer from 20,000 to 25,000 psi, and from 5 cycles to 7 cycles, respectively, did not effectively reduce the droplet size and PDI of AG-NEs. On the contrary, their droplet size and PDI tended to increase as seen in the open up parabolic shapes of the response surface plots in Figure 1a,b. This finding indicated that the values of pressures and number of homogenization cycles reached the over-processing conditions for the preparation of AG-NEs by a microfluidization method [16,22]. It could be explained that during the microfluidization process, the specific surface area of the droplets of AG-NEs was dramatically increased. In particular, in this study, concentrations of Tween 80 and lecithin, which were emulsifiers used in the formulation, were fixed at 8% *w*/*w* and 2% *w*/*w*, respectively. These concentrations of both emulsifiers were generally accepted to be enough to stabilize the new surface of the droplets because they could prevent coalescence of such oil droplets as seen in the promising stability of all AG-NEs, which was previously reported. 

However, when the pressure of the microfluidizer and number of homogenization cycles were further increased, the flow rate of the pre-emulsion of AG-NEs in the microfluidizer was more increased and caused higher collision rates of the pre-emulsion streams before generating nanoemulsion droplets. Consequently, the distribution of emulsifier molecules that involved breakage and coalescence of the droplets was more disrupted. Since the rate of collision of the droplets in the microfluidizer was higher than the adsorption rate of emulsifier molecules on the new droplet surfaces, coalescence of the droplets took place, and then the larger droplet size and broader droplet size distribution would be obtained [22].

### 3.4. Effects of Pressure and Number of Homogenization Cycles on the Zeta-Potential of AG-NEs

It is pertinent to indicate that AG-NEs prepared in this study had negative values of the zeta potential. This result arose from the negative charges from glycerol-phosphate head group of the constituents in lecithin [20] and the anion species in the mixed oils such as fatty acids [13] which could be ionized at the pH of AG-NEs prepared in this study (Table 2). All AG-NEs prepared in this study possessed low negative values of the zeta potential since a major emulsifier used, i.e., Tween 80, is a non-ionic surfactant that could be adsorbed rapidly at the droplet interface [21] and hindered the exposure of the anionic species consisting of the droplets [23]. 

Figure 1c depicted the response surface of the quadratic model of Equation (8). It showed that at the pressure of 15,000 to 20,000 psi, and a number of homogenization cycles of 3 to 5 cycles, the more negative values of the zeta potential of AG-NEs were obtained when the values of these parameters were increased. However, lesser negative values of zeta potential were obtained when AG-NEs were prepared at the pressure of 20,000 to 25,000 psi, and the number of homogenization cycles of 5 to 7 cycles. These findings indicated that over-processing conditions of microfluidization also affected the zeta potential values of AG-NEs, apart from the droplet size and PDI. It could be explained that upon the larger droplet size of AG-NEs was obtained at the over-processing conditions of microfluidization, the surface charge density of each droplet was declined [24,25]. These phenomena were consistent with the previous study on fish oil NE. It was reported that the zeta potential of the fish oil NEs tended to have less negative values when reaching the over-processing conditions of their production by using a microfluidization technique [26].

The production of AG-NEs via the microfluidizer at the pressure of 20,000 psi for 5 cycles of homogenization provided optimum values of droplet size, PDI and zeta potential of AG-NEs. This microfluidization condition was accepted as the optimum for the production of AG-NEs and would be used for the preparation of AG-NEs in further experiments. AG-NE prepared by using the optimized condition was called optimized AG-NE (Op-AG-NE). 

### 3.5. Preparation and Characterization of Optimized AG-NE and Blank NE

Optimized AG-NE (Op-AG-NE) and its NE base (Blank NE), which did not contain AG, were prepared by using the optimized condition. The basic physicochemical properties of Op-AG-NE, i.e., droplet size, PDI, zeta potential and pH, were 176.6 ± 1.8 nm, 0.332 ± 0.004, −11.78 ± 0.11 mV, and 5.7 ± 0.1, respectively. They were statistically comparable to droplet size, PDI, zeta potential and pH of Blank NE, which were 175.2 ± 1.0 nm (*p*-value = 0.308), 0.329 ± 0.004 (*p*-value = 0.374), −11.82 ± 0.12 mV (*p*-value = 0.708) and 5.6 ± 0.1 (*p*-value = 0.519), respectively. The results suggested that the addition of AG did not significantly alter these basic physicochemical properties of the NE base.

Drug entrapment efficiency (%EE) and drug loading capacity (%LC) of Op-AG-NE were 94.8 ± 0.8 and 0.32 ± 0.00, respectively. The result of EE indicated that most of the AG added into the formulation was entrapped in the oil droplets. However, the LC of Op-AG-NE was slightly low due to a limitation of AG solubility in the oil phase. It was found that the solubility of AG in the mixed oil was 0.12 ± 0.01 mg/mL. High concentrations of the oil mixture (20% *w*/*w*) and mixed emulsifier (10% *w*/*w*) were thus needed for preparation of 0.1% *w*/*w* AG-NEs in this study.

### 3.6. Morphology Observation of Op-AG-NE and Blank NE

**Figure 2:**
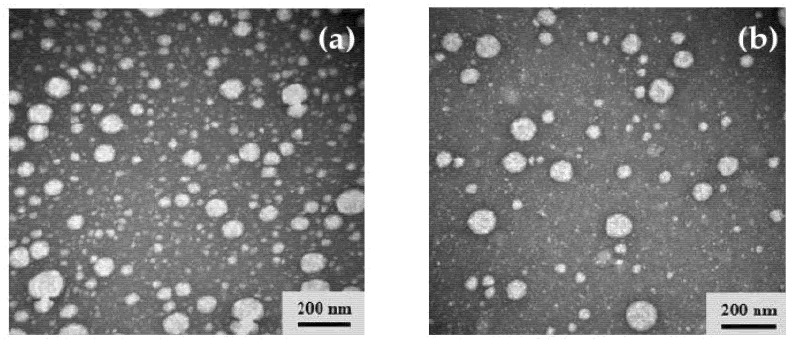
TEM micrographs: (**a**) Op-AG-NE and (**b**) Blank NE.

Morphology of Op-AG-NE and Blank NE were observed by transmission electron microscopy (TEM) technique. The micrographs shown in Figure 2a,b demonstrated that the droplet size of Op-AG-NE and Blank NE, respectively, were less than 200 nm with moderately broad droplet size distribution. These findings were consistent with the results from the measurement of drop size and PDI by a dynamic light scattering technique. In addition, the micrographs illustrated the identical spherical shape and droplet size of the internal phase of Op-AG-NE and Blank NE and evidenced that an addition of AG did not obviously affect the morphology and droplet size of the NE base.

### 3.7. FT-IR Spectroscopy Analysis 

**Figure 3:**
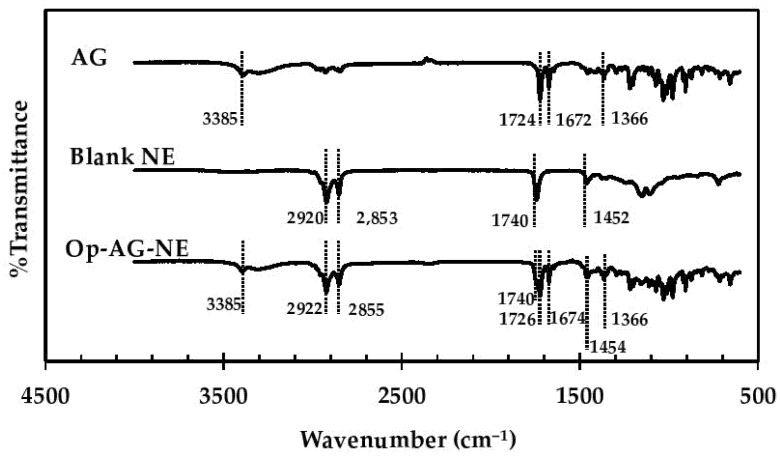
FT-IR spectra of AG, Blank NE and Op-AG-NE.

Interactions between AG and the ingredients of Op-AG-NE formulation were determined by using an FT-IR spectroscopy technique. FT-IR spectra of AG, Blank NE and Op-AG-NE shown in Figure 3 illustrated characteristic bands of the chemical structure of AG and the ingredients consisting in Blank NE and Op-AG-NE. The characteristic bands shown in the FT-IR spectrum of AG at a wavenumber of 1366 cm^−1^ represented vibration of –CH_3_. The bands at wavenumber 1672 cm^−1^ and 1724 cm^−1^ responded to stretching vibrations of C=O and the band at a wavenumber of 3385 cm^−1^ was assigned for stretching the vibration of hydrogen bonded in –O-H in the molecule of AG [27]. The band in the FT-IR spectrum of Blank NE at a wavenumber 1452 cm^−1^ responded to a bending vibration of C-H in the chemical structure of some ingredients in Blank NE, such as coconut oil [28]. The characteristic bands at a wavenumber of 1740 cm^−1^ was assigned for the C=O stretch of the ester bond between the fatty acid and the head group of phospholipids consisting of lecithin. The strong bands at wavenumbers of 2853 cm^−1^ and 2920 cm^−1^ also responded to the CH_2_ symmetric and symmetric stretch mode, respectively, in such phospholipid molecules [29]. 

The FT-IR spectrum of Op-AG-NE shown in Figure 3 pointed out that its FT-IR spectrum contained all the mentioned characteristic bands found in the FT-IR spectra of AG and Blank NE. Most of them appeared at the same wavenumber as previously described. However, trivial shifts of some characteristic bands were found. The characteristic bands of AG, those responded to stretching vibration of C=O, were shifted to 1674 cm^−1^ and 1726 cm^−1^. Meanwhile, the characteristic bands, those represented bending vibration of C-H, CH_2_ symmetric and symmetric stretch mode of some ingredients comprising of Blank NE, were shifted to 1454 cm^−1^, 2855 cm^−1^ and 2922 cm^−1^, respectively. These results indicated that AG was loaded to Op-AG-NE properly and there were some interactions such as hydrogen bonding and hydrophobic forces between the AG and NE base, which affected the release of AG from Op-AG-NE.

### 3.8. In Vitro Drug Release and Skin Permeation Study of Op-AG-NE 

Prior to the studies, content uniformity of AG-NE was determined by an HPLC technique. Results of analysis showed that AG contents in Op-AG-NE prepared in this study were 0.10 ± 0.00 g/100 g (*n* = 3). It was within a range of 90–100% of the labeled amount (0.10 g/100 g) and indicated that Op-AG-NE was prepared properly with homogeneity of AG content in the product. 

**Figure 4:**
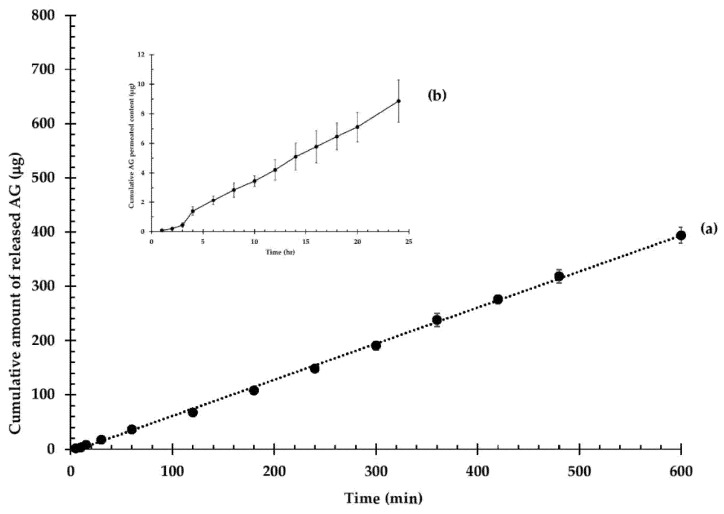
In vitro release profile and skin permeation of AG from Op-AG-NE: (**a**) cumulative amount of released AG and (**b**) cumulative AG permeated content (mean ± SD, *n* = 3).

The release profile of AG from Op-AG-NE was depicted in Figure 4a. It shows a continuous release of AG, which obeyed the zero-order release kinetic (r^2^ = 0.9985), with a rate of 0.66 ± 0.02 µg/min. The results implied that the AG release rate was solely controlled by a diffusion of AG from the droplets and was independent of the AG concentration. The constant release rate resulted in possible benefits of Op-AG-NE. It could prolong actions of AG in the skin tissues at least 10 h, thus, Op-AG-NE could lower the frequencies of administration.

The skin permeation profile of Op-AG-NE is presented in Figure 4b. It shows a non-steady state of skin permeation of AG at the first 3 h of the study, and then a steady state of skin permeation of AG as illustrated in a linear portion of the profile. A lag time of skin permeation of AG, which was estimated by extrapolating the linear portion to the axis of time, was 48.5 ± 2.5 min (0.81 ± 0.04 h). It indicated that a concentration gradient of AG that was released from Op-AG-NE in the skin reached an equilibrium at around 49 min after the experiment was performed. 

The linear portion of the permeation profile presented in Figure 4b was used for calculation of a flux of AG according to the Fick’s first law of diffusion. The result indicated that the flux of AG through the pig skin was 0.30 ± 0.03 µg/cm^2^∙h. It implied that around 0.30 µg of AG in Op-AG-NE could permeate through a unit cross section of the skin in an hour.

### 3.9. In Vitro Cytotoxicity Test of AG Solution, Blank NE and Op-AG-NE in the Normal Human Skin Fibroblasts 

The in vitro cytotoxicity test of AG solution, Blank NE and Op-AG-NE were per-formed in the human skin fibroblasts (HFF-1 cells) using an MTT assay. In this study, AG was dissolved in the cell culture medium containing DMSO to make various concentrations of AG solutions. 

Op-AG-NE was also diluted with the same vehicle based on the content of AG to prepare the test samples of those which contained the same concentration of AG as AG solutions. Meanwhile, Blank NE was diluted based on the content of Op-AG-NE used for preparation of the test samples. To avoid confusion, the concentration of all test samples used in this particular study was presented as “equivalent concentration”.

**Figure 5:**
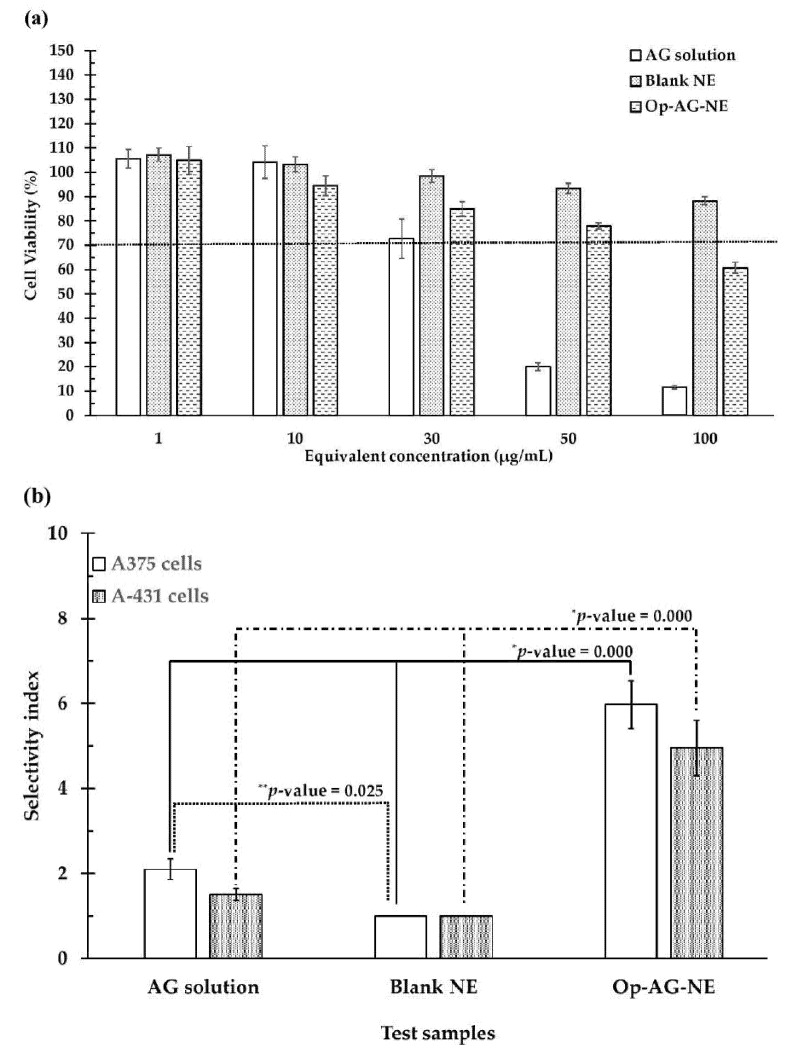
(**a**): Cell viability (%) of the HFF-1 cells; (**b**) Selectivity index of AG solution, Blank NE and Op-AG-NE in the A375 and A-431 cells (mean ± SD, *n* = 3) (* and ** represent significant difference at *p*-values < 0.05 for the comparison of selectivity index of AG solution in the cells and that of Op-AG-NE, and for the comparison of selectivity index of AG solution in the cells and that of Blank NE, respectively).

Figure 5a shows that the higher the equivalent concentrations of the test samples, the lower the CV of the HFF-1 cells. Since the CV of the HFF-1 cells incubated with Blank NE was more than 70% at entire equivalent concentrations (1–100 μg/mL), the Blank NE used in this study was not toxic to the HFF-1 cells [29]. 

AG solution exhibited a toxic effect on the HFF-1 cells (%CV of the cells was less than 70) at the equivalent concentration of 50 μg/mL and 100 μg/mL, while the toxicity of Op-AG-NE was found at the equivalent concentration of 100 μg/mL. This result was due to the cytotoxic activity of AG consisting of the formulation that could induce inhibition of cell proliferation [30,31] and cell death. Generally, the cytotoxic effect of AG was found in cancer cells; however, it might also be found in the normal cells when exposed to AG at high concentrations. This finding was consistent with the previous report by Suzuki et al. [30]. They found that AG was toxic to not only the human oral squamous cell carcinoma cells, but also the normal human oral cells at its high concentration. However, the inhibitory activities of AG was more selective in the human oral squamous cell carcinoma cells than the normal human oral cells. Therefore, AG was accepted as a promising cytotoxic agent against these oral cancer cells.

It is worth noting that the CV of the HFF-1 cells exposed to 100 μg/mL of an equivalent concentration of Op-AG-NE was much higher than that of the HFF-1 cells exposed to AG solution (around 5.23 folds). This finding suggested that Op-AG-NE was safe to the HFF-1 cells, more so than the AG solution. This result was from the controlled release effect of Op-AG-NE causing a gradual increase of AG concentration in the cells and thus delayed the cytotoxicity effect on the normal cells.

### 3.10. Determination of Cytotoxic Selectivity of AG Solution, Blank NE and Op-AG-NE in the Skin Cancer Cells 

Cytotoxic selectivity of the test samples were determined and presented as a selectivity index (SI). The results shown in Figure 5b elucidated that Op-AG-NE had a higher value of SI than that of the AG solution and Blank NE in the human malignant melanoma- (A375 cells) and non-melanoma cells (A-431 cells) at a *p*-value of 0.000 and 0.000, respectively. These findings implied that the cytotoxic activity of Op-AG-NE in the A375 and A-431 cells were more selective than that of AG solution and Blank NE; meanwhile, Op-AG-NE was safe to the normal human skin cells (HFF-1 cells). Apart from the effect of a controlled release of AG from Op-AG-NE, this result was due to the oil droplets of Op-AG-NE, an o/w NE, facilitated cellular uptake of AG via endocytosis into the A375 and A-431 cells. They thus prevented the cellular efflux of AG in both cancer cells, which was a self-protective mechanism of the cancer cells to reduce the cellular uptake of anti-cancer agents [32,33].

### 3.11. Apoptosis Analysis of the Skin Cancer Cells after Incubation with an AG Solution and Op-AG-NE

**Figure 6:**
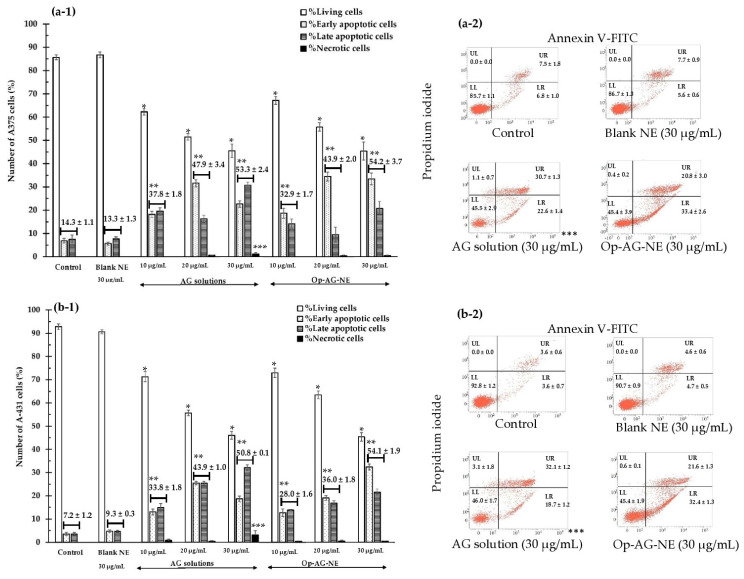
Apoptosis analysis: (**a-1**) and (**a-2**) in the A375 cells; (**b-1**) and (**b-2**) in the A-431 cells after incubation with Blank NE (30 µg/mL), AG solution and Op-AG-NE at various equivalent concentrations (mean ± SD, n = 3) (*, ** and *** represent significant difference at *p*-values < 0.05 when compared to number of the living cells, total apoptotic cells and necrotic cells of the controls, respectively).

Figure 6 shows the number of the A375 cells and A-431 cells to those exposed to the test samples, i.e., AG solution and Op-AG-NE for 24 h with a double stain of Annexin V-FITC and propidium iodide (PI). In this study, Blank NE, which was a base of Op-AG-NE without the addition of AG, was also used as a negative control at an equivalent concentration of 30 µg/mL.

The number of living cells (Annexin V-FITC-, PI-: LL) of the A375 cells and A-431 cells incubated with AG solution and Op-AG-NE are illustrated in Figure 6(a-1,b-1). They were significantly lower than that of the controls and that of the cells incubated with Blank NE (30 µg/mL) (*p*-values = 0.000 for all comparisons). Furthermore, they were markedly decreased when the equivalent concentrations of these test samples were increased. The findings indicated that the higher the equivalent concentrations of AG (in AG solution and Op-AG-NE), the higher the cytotoxic effect on both skin cancer cells. The results were consistent with the number of total apoptotic cells of the A375 cells and A-431 cells estimated by summation of numbers of the early apoptotic cells (Annexin V-FITC+, PI-: LR) and that of the late apoptotic cells (Annexin V-FITC+, PI+: UR). Their total numbers of apoptotic cells were much greater than that of the controls and the cells incubated with Blank NE (30 µg/mL) (*p*-values = 0.000 for all comparisons), and were much higher when the equivalent concentrations of the test samples were increased. They pointed out that the apoptosis induction activity of the AG solution and Op-AG-NE in the A375 and A-431 cells were in a dose-dependent manner. 

The numbers of the living cells, total apoptotic cells of the A375 and A-431 cells, which were incubated with Blank NE (30 µg/mL), were statistically comparable to those of the controls (*p*-values = 1.000 for all comparisons). This finding indicated that Blank NE could not exhibit cytotoxicity to the A375 and A-431 cells, although its highest equivalent concentration (30 µg/mL) was used. These results thus confirmed that cytotoxic activities of Op-AG-NE were from the activities of AG in the formulation. Liu and Chu [31] reported that AG could initiate the apoptosis process by activation of caspase-3 in the cells. It thus led to DNA fragmentation and cleavage of poly(adenosine diphosphate-ribose) polymerase. In addition, it increased the activity of c-Jun N-terminal kinases (JNKs) and p-p38 phosphorylation, which involved cell cycle G2/M phase arrest and apoptosis. Consequently, AG could inhibit cell growth and proliferation of the cells. 

Necrosis of both cells (Annexin V-FITC-, PI+: UL) were obviously found when they were incubated with an AG solution at a concentration of 30 μg/mL (the highest equivalent concentration) as presented in Figure 6(a-2,b-2). It was found that the number of necrotic cells of the A375 and A-431 cells after incubation with AG solution (30 μg/mL) were significantly higher than that of the controls and that of the cells incubated with Blank NE and Op-AG-NE (*p*-values = 0.000 for all comparisons). 

The results suggested that at an equivalent concentration of 30 µg/mL, AG solution exhibited strong cytotoxic effects on both skin cancer cells by induction of apoptosis and necrosis, which thus resulted in a rupture of the cell membrane, complete cell damage, and finally, spillage of the cell components into the environment [34]. Meanwhile, cytotoxicity activities of Op-AG-NE were still mainly from the induction of apoptosis. Therefore, controlled release of AG from Op-AG-NE also affected the cytotoxic effect of AG.

### 3.12. Investigation of Intracellular Tryrosinase Inhibitory Activity of Op-AG-NE

As previously mentioned, UV irradiation could induce not only skin cancer but also skin pigmentation via activation of tyrosinase activity. To elucidate the activity of Op-AG-NE for inhibition of intracellular tyrosinase, the A375 cells were incubated with the test samples as listed: AG solution, Op-AG-NE, Blank NE (a negative control) and kojic acid (a positive control) at an equivalent concentration of 6 μg/mL for 72 h to avoid their cytotoxic effect. 

**Figure 7:**
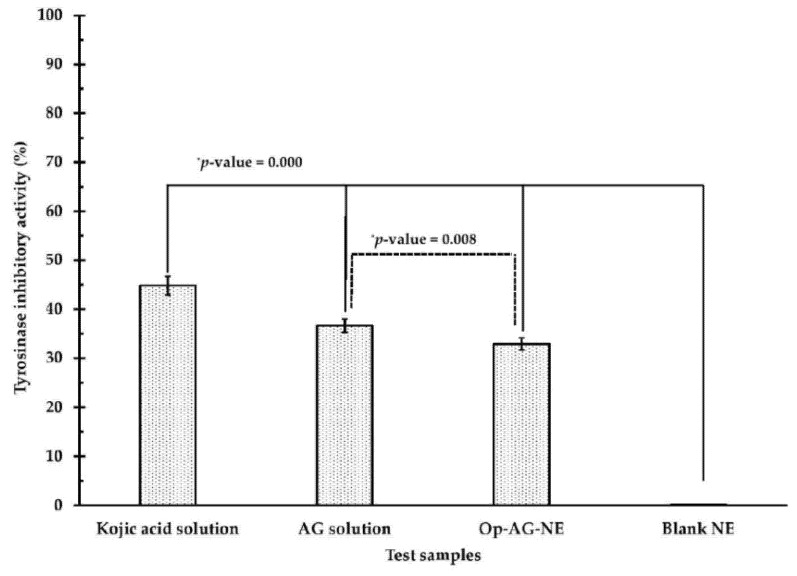
Intracellular tyrosinase inhibitory activity (%) of the test samples in the A375 cells (mean ± SD, *n* = 3) (* significantly different at a *p*-value < 0.05).

Figure 7 indicates that all test samples could markedly reduce the activity of intracellular tyrosinase of the A375 cells except for Blank NE. Among these test samples, kojic acid solution, which was a positive control, exhibited a stronger inhibitory activity against intracellular tyrosinase of A375 cells than that of AG solution, Op-AG-NE and Blank NE, respectively, at *p*-values of 0.000 (for all comparisons). Since Blank NE did not show the inhibitory activity against intracellular tyrosinase of A375 cells, the inhibitory activity of Op-AG-NE against tyrosinase came from the effect of AG, consisting of the product. 

Zhu et al. [7] found that AG could inhibit intracellular tyrosinase via down-regulation of MITF (microphthalmia-associated transcription factor) expression, and consequently, resulting in lower melanin synthesis in the cells. Therefore, Op-AG-NE could be used as a delivery system of AG for anti-skin pigmentation. However, tyrosinase inhibitory activity of Op-AG-NE in the A375 cells was significantly weaker than that of AG solution at a *p*-value of 0.008. This finding was due to the fact that controlled release of AG from the droplets of Op-AG-NE retarded the accumulation rate of AG content in the cells, leading to a weaker inhibitory activity against intracellular tyrosinase in the A375 cells. 

### 3.13. Effect of Op-AG-NE on UVB Irradiation-Induced Skin Pigmentation and Skin Damage in the Rats

Op-AG-NE used in this section was modified in its viscosity by adding methylcellulose (2% *w*/*w*) into the formulation to prevent the product leaking out of the applied skin. The obtained product called “Op-AG-NE gel” still contained AG content at the same concentration as Op-AG-NE (0.1% *w*/*w*) before incorporation of methylcellulose. 

The viscosity of Op-AG-NE and Op-AG-NE gel measured at a shear rate of 300 s^−1^ were 10.2 ± 1.0 mPa.s and 1009.4 ± 4.3 mPa.s, respectively. The flow behavior of Op-AG-NE was altered from a Newtonian to a pseudoplastic behavior after methylcellulose was incorporated. This particular flow behavior of Op-AG-NE gel provided benefits for application on the skin because it could be applied easily with its lower viscosity when being applied on the skin. However, it could stay on the skin without leaking due to its high viscosity after application on the skin. In this study, Blank NE gel, which was a NE gel base of Op-AG-NE gel without addition of AG, was also used as a negative control.

**Figure 8:**
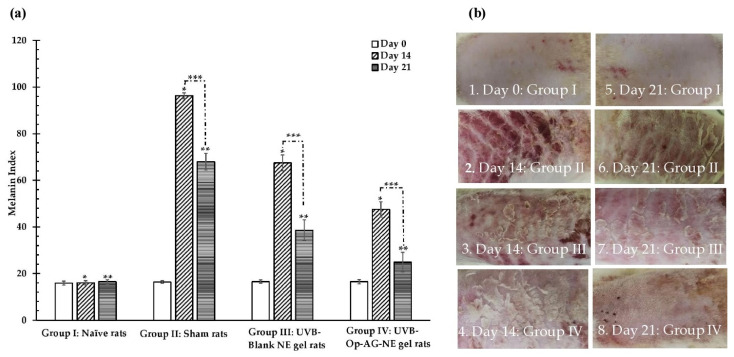
(**a**) Melanin Index (mean ± SD, *n* = 10) and (**b**) macroscopic skin appearance of the UVB-exposed rats’ skin (*, ** and *** were significantly different at a *p*-value < 0.05).

The melanin index and macroscopic appearances of the rats’ skin were observed on day 0 before starting the experiment as shown in Figure 8(a,b-1), respectively. They were followed up on day 14 and day 21 of the experiment period. It was found that the melanin index and skin appearance of the rats’ skin in Group I (naïve rats) did not significantly change during this 21-day experiment as illustrated in Figure 8(a,b-5) and were comparable to those of the rats’ skin in Group II, III and IV at day 0. On the contrary, increase in the melanin index and dramatic changes of the skin appearances, i.e., erythema and epithelial hyperplasia, were found in the rats of Group II (sham rats), III (UVB-Blank NE gel rats) and IV (UVB-Op-AG-NE gel rats) on day 14, as seen in Figure 8(a,b-2,b-3,b-4), respectively. This finding indicated that UVB irradiation strongly induced the melanin synthesis and skin damage in the rats’ skin [6,7]. In particular, the rats in Group II had the highest melanin index with obvious skin erythema and epithelial hyperplasia because they did not receive any treatment during the study. 

On day 14, the mean value of melanin index of the rats in Group IV was significantly lower than that of the rats in Group II and III at *p*-values of 0.000 and 0.000, respectively. In addition, changes in skin appearance of the rats in Group IV shown in Figure 8(b-4) were also less than those of the rats in Group II and III as illustrated in Figure 8(b-2,b-3), respectively. These results implied that Op-AG-NE gel could reduce the effect of UVB irradiation-induced skin pigmentation and could protect the skin from UVB irradiation. 

Figure 8 pointed out that the melanin index and changes of macroscopic appearance of the rats’ skin in Group III were less than those of the rats in Group II; however, they were higher than those of the rats in Group IV. These results suggested that NE base gel also had activities against UVB irradiation-induced skin damage activities because of the activities of the ingredients, such as the mixed oil that consisted of the formulation. However, its activities were still lower than those of Op-AG-NE gel used in the rats in Group IV. Thus, it could be concluded that AG was the active compound which mainly exhibited activities against UVB irradiation-induced skin damage. This finding was in agreement with the previous studies by Zhan et al. [6], Zhu et al. [7] and Yen et al. [12], those that reported that AG possessed inhibitory activities against UVB irradiation-induced oxidative stress, pigmentation and inflammation in skin as well as photoaging. 

On day 21, the melanin index of the rats in Group II, III and IV were lower than that of the rats in their same group on day 14 (*p*-values = 0.000 for all comparisons). Recovery of the skin appearance of the rats in these groups could be also observed as seen in Figure 8(b-6,b-7,b-8). This finding was due to the rats in these groups not being exposed to UVB radiation since day 14. Amongst these three groups, the melanin index of the rats in Group IV on day 21 was lower than that of the rats in Group II and III at a *p*-value of 0.000 and 0.000, respectively. Their skin appearances shown in Figure 8(b-8) were also better than those of the rats in Group II and III, as presented in Figure 8(b-6,b-7), respectively. The epidermal hyperplasia as well as scabs of the burn wounds from UVB irradiation were less found in the rats of Group IV. It could be explained that AG in Op-AG-NE gel could protect the skin from UVB irradiation and exhibited healing effect on the rats’ skin those were damaged by UVB irradiation. Therefore, Op-AG-NE gel could be accepted for management of skin pigmentation and skin damage induced by UVB irradiation.

## 4. Conclusions

Physicochemical properties, i.e., droplet size, PDI and zeta potential, of AG-NEs prepared by microfluidization technique, were affected by pressure and number of homogenization cycles. The relationships between pressure and number of homogenization cycles versus values of droplet size, PDI and zeta potential of AG-NEs could be described by quadratic models, which were obtained from the face-centered central composite design (FCCD). The increase in pressure and number of homogenization cycles led to small droplet size, low PDI and a more negative value of zeta potential of AG-NEs. Unfortunately, an extreme increase in the pressure and number of homogenization cycles resulted in the increase in droplet size, PDI and less negative value of zeta potential. The optimized AG-NE (Op-AG-NE) prepared by using a pressure of 20,000 psi for 5 homogenization cycles was selected as a representative of AG-NEs for investigation of the bioactivities. Op-AG-NE had a droplet size, PDI, zeta potential and pH of 176.6 ± 1.8 nm, 0.332 ± 0.004, −11.78 ± 0.11 mV and 5.7 ± 0.1, respectively, with an EE (%) and an LC (%) of 94.8 ± 0.8 and 0.32 ± 0.00, respectively. The release kinetic of AG from Op-AG-NE was in agreement with the zero order kinetic. Op-AG-NE prepared in this study was not toxic to the normal skin fibroblasts (HFF-1 cells) at the equivalent concentrations of 1–50 μg/mL. It had higher values of selectivity index than that of AG solution and Blank NE and exhibited cytotoxicity to the human malignant melanoma- (A375 cells) and non-melanoma cells (A-431 cells), mainly via the induction of cell apoptosis. Op-AG-NE could inhibit the activity of intracellular tyrosinase in the A375 cells properly. However, its activity was lower than kojic acid solution and AG solution. The results also indicated that Op-AG-NE could reduce UVB irradiation-induced skin pigmentation and damage in the rats properly. It reduced the values of melanin index of the rats exposed to UVB radiation and healed the rats’ skin after exposure to UVB radiation. 

Therefore, Op-AG-NE had benefits for transdermal applications, in particular, for the treatment of skin disorders from exposure to UVB radiation. It is a promising product for using in further clinical studies to investigate its efficacy and molecular mechanisms in the treatment of melanoma-, non-melanoma skin cancers, skin pigmentation as well as skin damage from UV irradiation.

## Figures and Tables

**Table 1:** Actual values and coded values of factors and responses for the FCCD.

AG-NE Number	Actual Values of Factors		Coded Values of Factors (*a*, *b*) **	Responses
	Pressure (psi) (*a*) *	Number of Homogenization Cycles (*b*) *		
AG-NE1	15,000	3	(−, −)	1. Droplet size 2. PDI 3. Zeta potential
AG-NE2	25,000	3	(+, −)	
AG-NE3	15,000	7	(−, +)	
AG-NE4	25,000	7	(+, +)	
AG-NE5	15,000	5	(−α, 0)	
AG-NE6	25,000	5	(+α, 0)	
AG-NE7	20,000	3	(0, −α)	
AG-NE8	20,000	7	(0, +α)	
AG-NE9	20,000	5	(0, 0)	
AG-NE10	20,000	5	(0, 0)	
AG-NE11	20,000	5	(0, 0)	
AG-NE12	20,000	5	(0, 0)	

* The letter *a* and *b* represent pressure and number of homogenization cycles, respectively. ** The symbols are as listed; (−), (0), (+), (−α), and (+α) were coded values of factors at various levels, i.e., a low level, a center point, a high level, a low level of axial point, and a high level of axial point, respectively.

**Table 2:** Observed values and mean of predicted values of the responses at 95% confidence interval, and pH of AG-NEs (mean ± SD, *n* = 3).

AG-NE Number	Code of (*a*, *b*) *	Observed Values of the Responses			Mean of Predicted Values of the Responses			pH
		Droplet Size (nm)	PDI	Zeta Potential (mv)	Droplet Size (nm)	PDI	Zeta Potential (mv)	
AG-NE1	(−, −)	250.1 ± 1.0	0.394 ± 0.003	−8.04 ± 0.02	247.9	0.413	−8.32	5.6 ± 0.1
AG-NE2	(+, −)	207.5 ± 2.3	0.306 ± 0.004	−9.65 ± 0.05	208.9	0.303	−9.57	5.7 ± 0.2
AG-NE3	(−, +)	219.1 ± 2.7	0.764 ± 0.004	−8.78 ± 0.03	220.5	0.753	−8.78	5.6 ± 0.1
AG-NE4	(+, +)	182.0 ± 2.5	0.644 ± 0.003	−10.04 ± 0.07	181.5	0.643	−10.02	5.6 ± 0.1
AG-NE5	(−α, 0)	220.4 ± 1.8	0.475 ± 0.005	−10.38 ± 0.05	221.2	0.466	−10.10	5.7 ± 0.2
AG-NE6	(+α, 0)	183.0 ± 4.2	0.352 ± 0.001	−11.23 ± 0.06	182.2	0.356	−11.34	5.6 ± 0.1
AG-NE7	(0, −α)	202.7 ± 2.7	0.304 ± 0.004	−10.18 ± 0.05	203.5	0.287	−9.99	5.6 ± 0.1
AG-NE8	(0, +α)	177.0 ± 2.2	0.615 ± 0.003	−10.42 ± 0.03	176.1	0.627	−10.44	5.6 ± 0.1
AG-NE9	(0, 0)	178.4 ± 4.0	0.354 ± 0.003	−11.62 ± 0.04	176.8	0.340	−11.76	5.7 ± 0.1
AG-NE10	(0, 0)	174.3 ± 1.0	0.324 ± 0.003	−11.52 ± 0.04	176.8	0.340	−11.76	5.6 ± 0.1
AG-NE11	(0, 0)	177.8 ± 2.8	0.343 ± 0.001	−11.82 ± 0.05	176.8	0.340	−11.76	5.7 ± 0.1
AG-NE12	(0, 0)	176.7 ± 2.1	0.332 ± 0.002	−11.92 ± 0.06	176.8	0.340	−11.76	5.6 ± 0.1

* The letter *a,* and *b* represented pressure and number of homogenization cycles, respectively. The symbols as listed; (−), (0), (+), (−α), and (+α) were coded values of factors at various levels, i.e., a low level, a center point, a high level, a low level of axial point, and a high level of axial point, respectively.

## Data Availability

Data are available on request due to privacy restrictions.
